# Sustainable Extraction of Fresh Banana Inflorescence by ASE: Optimization and Characterization of Anthocyanin Rich Extracts by LC-UV-MS/MS

**DOI:** 10.3390/foods14081299

**Published:** 2025-04-08

**Authors:** Nuwanthi Senevirathna, Morteza Hassanpour, Ian O’Hara, Azharul Karim

**Affiliations:** 1 School of Mechanical, Medical and Process Engineering, Faculty of Engineering, Queensland University of Technology, Brisbane 4000, Australia; nuwanthi.senevirathna@hdr.qut.edu.au (N.S.); m.hassanpour@qut.edu.au (M.H.); i.ohara@qut.edu.au (I.O.); 2 Centre for Agriculture and the Bioeconomy, Faculty of Science, Queensland University of Technology, Brisbane 4000, Australia; 3 ARC Centre of Excellence in Synthetic Biology, Queensland University of Technology, Brisbane 4000, Australia; 4 ARC Industrial Transformation Training Centre for Bioplastics and Biocomposites, Queensland University of Technology, Brisbane 4000, Australia

**Keywords:** accelerated solvent extraction, ASE, antioxidants, anthocyanins, banana inflorescence, phenolics

## Abstract

Sustainable and environmentally friendly extraction methods for natural bioactive compounds are gaining significant attention in the food, beverage, and nutraceutical industries. Among these bioactive compounds, anthocyanins, which are potent antioxidants, have garnered particular interest due to their health-promoting properties. Banana inflorescence, an underutilized agricultural by-product, is a rich source of bioactive compounds. However, the extraction of bioactive compounds is often energy-intensive, which raises concerns about environmental sustainability. Accelerated solvent extraction (ASE) has emerged as an efficient and less energy-consuming method for isolating these compounds. This study investigates the optimization of ASE for the extraction of phenolic compounds, including anthocyanins, from fresh banana inflorescence. The effect of extraction parameters, including temperature (60, 80, and 100 °C), solvent type (water, ethanol, methanol), and solvent composition (50% ethanol + 50% water, 75% ethanol + 25% water, 100% ethanol, 50% methanol + 50% water, 75% methanol + 25% water, 100% methanol, water), on the extraction efficiency was evaluated. The results showed that the most effective extraction conditions were 75% methanol + 25% water at 100 °C, yielding the highest concentrations of total phenolics (1239.58 ± 20.83 mg/100 g), antioxidant activity (2.21 ± 0.03 mg/mL), and anthocyanins (22.82 mg ± 1.91/100 g). LC-UV-MS analysis revealed three primary anthocyanidins: cyanidin-3-rutinoside, delphinidin-3-rutinoside, and petunidin-3-rutinoside. These findings suggest that banana inflorescence, an agricultural waste product, can be efficiently utilized as a source of bioactive compounds using ASE, contributing to sustainable practices in the food and nutraceutical industries. The optimized extraction process provides a promising approach for the valorization of banana inflorescence, enhancing its potential as a functional ingredient in food products.

## 1. Introduction

Fruits and vegetables are rich in bioactive compounds and play an important role in human well-being owing to their potential for combating major non-communicable diseases specifically in the prevention of cardiovascular diseases and chemoprotective abilities [1]. Generally, bioactive compounds exist in natural food products as phytochemicals such as phenols, flavonoids, terpenoids, triterpenes, steroids, etc. These compounds can be utilized for functional food applications [2], pharmaceutical production, and nutraceutical industries. Functional food development is an innovative emerging technology in the food industry worldwide [3]. As it can positively support targeted functions in the body [2]. The by-products from the production and processing of many fruits and vegetables are also rich in bioactive compounds.

Banana inflorescence is a by-product of banana cultivation [4], usually considered a waste [5]. There is a high demand for bananas around the world and there are more than a thousand banana cultivars and varieties with diverse nutritional and bioactive properties. However, commercial production is dominated by Cavendish. As the banana is one of the most widely cultivated tropical crops worldwide, bananas are grown throughout the year in many countries, resulting in the generation of large quantities of banana by-products which account for nearly 80% of the biomass of banana crops [6]. Banana inflorescence is an edible by-product which is generated at a rate of 300 kg/Ha [7,8]. Banana inflorescence is usually either left to decompose in the banana field [7] or goes to a landfill [9] contributing to methane generation and greenhouse gas production.

Banana inflorescence has proven medicinal benefits, functional properties, and potential industrial applications in food and drug (health) industries [7,9,10,11]. It is a prominent source of bioactive compounds as it is rich in phytochemicals such as phenolic compounds, flavonoids, steroids, triterpene, alkaloids, saponins, tannins, and essential microelements [12]. Importantly, the banana inflorescences possess high antioxidant activity which aids in preventing non-communicable diseases. Banana inflorescence has been shown to have antibacterial, antidiabetic, anticancer [13], antimicrobial, antioxidative, and anti-inflammatory properties [14]. Previous studies have shown that banana inflorescence has abundant anthocyanins which is a natural pigment having anticancer and antimicrobial potential [15,16]. The pharmacological behaviours of the banana inflorescence have gained much scientific attention as they offer promising potential in innovative pharmaceutical and nutraceutical products.

Therefore, determining the presence of bioactive compounds, their availability, composition, and quality analysis is crucial for planning and implementing innovative industrial approaches. The quantity and quality of bioactive compounds depend on the banana variety (genetics), growing conditions, geographical differences, and method of processing and extraction. To date, many studies have determined the bioactive compounds available in dried *Musa paradisiaca* inflorescence extracted using conventional extraction methods [17,18]. However, no study has examined the bioactive compounds in the fresh banana inflorescence of *Musa accuminata* Cavendish.

Processing methods and efficiency have a significant effect on the extraction of bioactive components. Reduction in the bioactive properties of banana flour has been reported in the literature during processing steps [19]. The bioactive properties of fresh banana inflorescence may also diminish during the processing steps such as drying. Therefore, appropriate drying of fresh banana inflorescence is critical. This study aimed to investigate the bioactive properties of fresh banana inflorescence with the aim of saving energy and processing time, which directly aligns with the United Nations’ sustainable development goals.

The yield of bioactive compounds varies depending on the method of extraction [20]. Extraction of phytochemicals from banana inflorescence using Soxhlet [18], maceration, and overnight soaking with solvents [21] such as ethanol [22,23] are the most widely used extraction methods in the literature. However, these methods have significant drawbacks, including lengthy processes, high solvent consumption, degradation of bioactive compounds, and the requirement for clean-up and concentration before the chromatographic analysis.

The accelerated solvent extraction (ASE), also known as the Pressurized Liquid Extraction (PLE) technique, extracts the analytes in a closed and inert dark environment within a short period using minimal amounts of solvents [24]. Dark inert conditions are highly recommended for most bioactive compounds as they are photosensitive [25]. It is a highly efficient and advanced technique for the extraction of bioactive compounds, particularly phenolic compounds and anthocyanins, compared to conventional methods such as maceration and Soxhlet extraction. ASE operates under elevated temperature and pressure, significantly enhancing mass transfer and solubility, leading to higher extraction yields within a shorter duration. Unlike Soxhlet extraction, which involves prolonged solvent reflux and the potential thermal degradation of heat-sensitive compounds, ASE allows for precise temperature control, minimizing degradation while maximizing recovery. Additionally, ASE requires significantly lower solvent volumes than both Soxhlet and maceration, improving sustainability and reducing environmental impact. The automation and reproducibility of ASE further enhance its efficiency, ensuring consistent results with minimal manual intervention, whereas maceration and Soxhlet extractions are time consuming, solvent intensive, and often yield lower extraction efficiencies. Moreover, ASE’s controlled conditions facilitate selective extraction, reducing the co-extraction of unwanted compounds that are often present in Soxhlet extraction. Given these advantages, ASE emerges as a superior extraction method for phenolic and anthocyanin-rich bioactive compounds, offering improved efficiency, reduced solvent usage, and enhanced compound stability, making it a preferred choice for both research and industrial applications.

A shorter extraction time is also an added advantage of ASE as it can reduce the degradation of bioactive compounds during the processing steps. Less solvent consumption is another benefit, which is a sustainable and green approach that saves both energy and other resources. Many scientific studies have shown that ASE is superior to other extraction methods in relation to bioactive compound yield and quality [26,27].

ASE has been used for the extraction of natural bioactive compounds from other food products such as grapes, apples [27], soybean, ginger [28], flaxseed, Tahiti lime [29], and sweet potato [25]. However, to the best of our knowledge, this is the very first investigation of the bioactive composition of fresh banana inflorescence extraction using ASE. The mechanism of extraction of fresh banana inflorescence using ASE [30] is an innovative study that highlights the sustainable utilization of waste by-products as valuable food ingredients in an eco-friendly manner.

In the present study, the optimization of bioactive compounds extraction from banana inflorescence was conducted using ASE from *Musa cavendish* banana inflorescence. This research endeavoured to address the dual challenge of optimizing extraction efficiency while adhering to energy-efficient and environmentally sustainable practices. The green approach entails the exploration and refinement of extraction parameters such as the solvent type, solvent concentration, and temperature, with the overarching goal of minimizing resource consumption and waste generation. These findings contribute to knowledge in the field of banana inflorescence by broadening the understanding of the bioactive compounds available in fresh banana inflorescence, the bioactive compounds yield, their retention, preservation, and degradation with different solvents and temperatures.

The outcomes of this study are poised to contribute valuable insights to the field of natural product extraction and to pave the way for a more environmentally harmonious approach to harnessing the potential benefits of bioactive compounds from banana inflorescence.

## 2. Materials and Methods

### 2.1. Banana Inflorescence Samples

**Figure 1:**
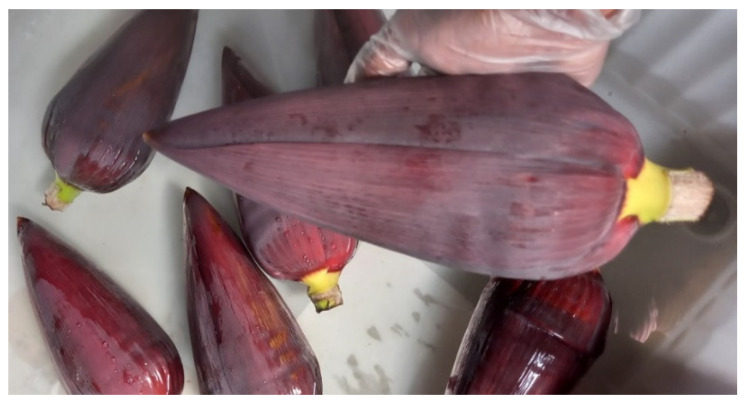
Debelling stage of banana inflorescence.

Fresh Cavendish banana inflorescence was collected from various banana farms in Wamuran, Queensland, Australia. Geographical differences can significantly impact the chemical composition and antioxidant properties of banana inflorescence samples. Samples from multiple locations in the same region at the same harvesting times were collected to mitigate the variability. The samples were rigorously selected to maintain uniformity, featuring lengths of 30 cm to 34 cm and weights between 1.00 kg and 1.4 kg. The collection of inflorescences at the debelling stage (the process of removing banana inflorescence from the banana bunch after the opening of the last bunch of fruits) was chosen for this study (Figure 1). This is the stage where banana inflorescences are usually slashed off at the farms.

### 2.2. Chemicals

All chemicals used in this research were of analytical grade or analytical standards. All chemicals were purchased from Sigma Aldrich, Castle hill, NSW, Australia. Distilled water was used for the extraction process and LC_MS grade water (LiChrosolv^®^ water, Merck KGaA, Darmstadt, Germany) for LC_MS analytical procedures. HPLC-grade ethanol and methanol were purchased from Thermofisher Scientific, Brisbane, Australia, for extraction purposes. Folin–Ciocalteau reagent, gallic acid, ascorbic acid, 2,2-diphenyl-1-picrylhydrazyl (DPPH), sodium carbonate, and Cyanidin-3-glucoside were also purchased.

### 2.3. Sample Preparation

**Figure 2:**
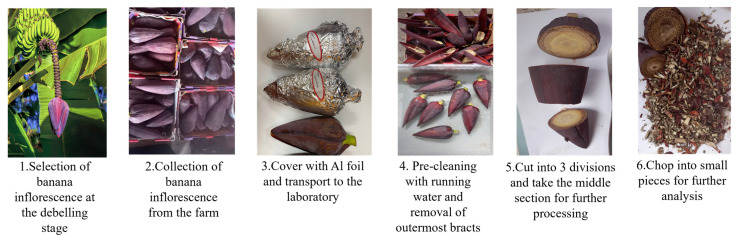
Sample preparation procedure.

Fresh Cavendish banana inflorescence samples at the debelling stage were collected and transported to the laboratory for experimental purposes. To prevent heat stress and photodegradation of the bioactive compounds, the debelling process was conducted in the evening. Additionally, the inflorescences were shielded with black polythene and promptly transferred to the laboratory within a day to minimize the impact of external factors such as heat, light, and transport stress. Upon arrival at the laboratory, the inflorescences were cleaned by immersing them in a clean water bath to remove surface impurities and contaminants. Subsequently, approximately five to six outer discoloured bracts, which had been extensively exposed to sunlight in the field, were removed to further clean the inflorescence. For this study, both bracts and male flowers were utilized, as opposed to previous studies that focused solely on separated banana bracts. Given that male inflorescence also serves as a promising source of bioactive compounds, our study was designed to encompass the entire banana inflorescence without separation. The cleaned inflorescences were divided into three equal portions, and the middle section was selected for further processing. Using a sharp knife, the selected middle section of the inflorescence was cut into approximately 0.5 cm × 0.5 cm pieces. The pre-cut samples underwent extraction on the same day. The sample preparation process is illustrated in Figure 2.

### 2.4. Experimental Process

**Figure 3:**
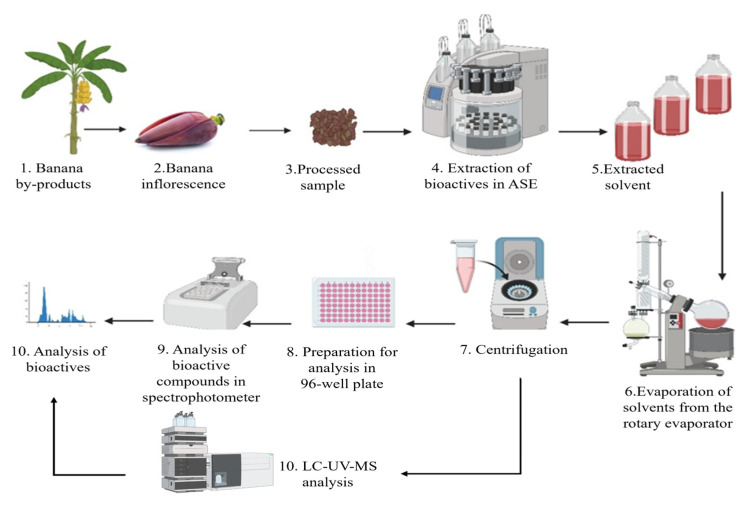
Experimental design.

Precut fresh inflorescences were subjected to extraction by an accelerated solvent extractor (Dionex ASE 350 Thermofisher Scientific, Waltham, MA, USA) at three different temperatures, 60 °C, 80 °C, and 100 °C, using ethanol and methanol at three different concentrations in water, 50, 75, and 100% (*v*/*v*). The extract was filtered, concentrated, and subjected to antioxidant and total phenolic content assays. The optimum conditions were identified for the extraction of bioactive compounds from the banana inflorescence. The extract was further analyzed for its anthocyanin profile via LC_MS analysis (Figure 3). Analyses were carried out in triplicate.

### 2.5. Physical Properties of Banana Inflorescence

#### 2.5.1. Morphology

Individual fresh banana inflorescences (***n*** = 100) were randomly chosen, and the length and width were measured. The weight of whole inflorescences was measured using a laboratory scale (A&D-GX-6100). The colour of the banana inflorescence was measured using a colourimeter (Konica Minolta Cr 10 plus, Tokyo, Japan) and converted to a HEX (Hue-Excitation) value using a nix sensor 2.0 software. The colourimeter was precisely calibrated using a standard white reference tile prior to sample analysis. According to the colourimeter, the L axis stands for lightness. The redness to greenness was presented on the a-axis. The b*-value explained a change in colour from yellow to blue.

Chroma (C*) refers to the intensity, saturation, or purity of a colour. A higher chroma value represents a more intense and saturated colour, while a lower value indicates a more muted or neutral tone. Colourimeters measure chroma by analyzing how light interacts with a sample, quantifying its colour intensity based on absorbance or reflectance at specific wavelengths. Chroma (C*) is calculated from the a* and b* values according to the calculation.

$$Chroma\left(C*\right)=\sqrt{{a*}^{2}+{b*}^{2}}$$

Hue $\left(H^{{}^{\circ}}\right)$ angle refers to the colour perception representing the dominant colour of a sample. It is calculated by using the a* and b* colourimetric coordinates.

$$\mathrm{Hue}(H^{{}^{\circ}})=\tan-1\frac{b*}{a*}$$

#### 2.5.2. Moisture Content

The moisture content of fresh banana inflorescence was measured by oven drying according to the AOAC method 977.11. Briefly, 10 g of fresh samples were placed in a convective oven (Labec, Marrickville, Australia) in crucibles maintained at 105 °C until a constant weight was reached and the moisture content was calculated based on the weight loss against the raw sample.

$$Total\;moisture\;content\;\%=\frac{Fresh\;weight\;\left(g\right)-Dry\;weight\;\left(g\right)}{Fresh\;weight\;\left(g\right)\;}\times 100$$

#### 2.5.3. Ash Content

The ash content was determined according to the AOAC method 923.03. The banana inflorescence was placed in cleaned crucibles for the determination of ash content. Ash content was determined gravimetrically, incinerated in a Carbolite Gero muffle furnace at 550 °C overnight.

$$Ash\;content\;\%=\frac{Weight\;of\;residue\;\left(g\right)}{Weight\;of\;fresh\;sample\;(g)\;}\times 100$$

#### 2.5.4. Microstructure

The fresh banana inflorescence of the Cavendish cultivar was cut into small pieces as described above in the sample preparation section. The surface characteristics and microstructure of fresh samples were examined under a light microscope (Leica-DFC490-M125 Stereo microscope, Leica Microsystems, Wetzlar, Germany) at 5000× magnification.

### 2.6. Extraction

The ASE 350 Dionex (Thermo Fisher Scientific, Waltham, MA, USA) extractor connected to N2 gas was utilized for this study as per the method explained by Azian et al. [28], with some modifications.

The extraction process was performed at a pressure of 1500 psi, 60 s of purging time per cycle, and a flush volume of 60%. The extraction duration was divided into 3 cycles, each lasting 7 min, with an additional 5 min preheating period. Stainless steel extraction cells of 36 mL were used. The bottom of each cell was lined with ASE filter glass fibre (34-thermo scientific grade) before loading the samples and 10 g of fresh banana inflorescence was added to each cell. Then, the extraction cells were filled up to 75% by adding 5.0 g of diatomaceous earth-DE (Thermo Fisher Scientific, P/N 062819) and thoroughly mixed with the fresh sample.

**Figure 4:**
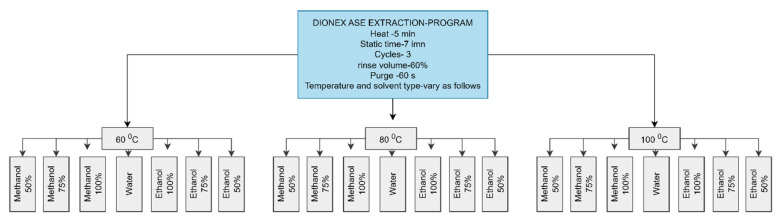
ASE diagram of bioactive compounds of fresh banana inflorescence.

The extractions were carried out using three solvents, water, methanol, and ethanol, at temperatures of 60 °C, 80 °C, and 100 °C according to a similar study conducted using ASE with slight modifications [29]. The selection of the temperatures (60 °C, 80 °C, and 100 °C) for accelerated solvent extraction (ASE) was for optimizing the efficiency and effectiveness of extracting bioactive compounds from plant matrices. Different temperatures can enhance the solubility and diffusion rates of bioactive compounds in the solvent, with higher temperatures generally increasing solubility and leading to more efficient extraction [31]. The chosen temperatures ensure the stability of the bioactive compounds, avoiding degradation at higher temperatures while ensuring sufficient extraction at lower temperatures. This range allows for the assessment of extraction kinetics, determining the optimal temperature for maximum yield. At the end of extraction, the volume of the extract was adjusted to 100 mL in a volumetric flask with the same extract solvent. The extract was then filtered using sterile 0.22 μm PTFE syringe filters before analysis. The filtered extracts were stored in a refrigerator at −4 °C for 3–4 days before the analysis. The final extract was used to determine the total antioxidant content and total phenolic content. The experiment was carried out with 21 different solvent and temperature combinations as illustrated in Figure 4.

### 2.7. Bioactive Properties of Extracts

The extracts of fresh banana inflorescence were then analyzed for their bioactive composition. The phenolic content, total antioxidant activity, and total anthocyanin content were studied. The anthocyanin profile of the best extract was further analyzed using LC-MS.

#### 2.7.1. Total Phenolic Content (Folin–Ciocalteau Method)

The total phenolic content was measured via the Folin–Ciocalteau method [32] with some modifications. Folin–Ciocalteau regent (2N) was used for this experiment. Briefly, 790 µL of distilled was added to 10 µL of extract prepared in 1.5 mL Eppendorf tubes. Thereafter, 100 µL of Folin–Ciocalteau solution was added, and the tubes were stood at room temperature for 5–8 min. Then, 150 µL of 20% sodium carbonate was added to each tube and mixed well. Finally, the samples were incubated at room temperature (20 °C) under dark conditions for 2 h. After incubation, 200 µL of aliquots from each tube were placed on a 96-well microtitration plate and the absorbance was read at 765 nm using a multimode microplate reader (Synergy biotek HTX). Samples were read against a gallic acid standard curve and results were expressed as gallic acid equivalent (mg GAE/100 g sample of dry weight). The results were analyzed using Gen 5.3 software.

#### 2.7.2. Total Antioxidant Activity (DPPH Analysis)

DPPH free radical scavenging activity was used to determine the total antioxidant activity of banana inflorescence as per the method explained by Yu [33] with some alterations. Briefly, 100 µL of 2,2-diphenyl-1-picryhydrazyl (DPPH) solution was added to 100 µL sample/standard/blank, the mixture was incubated at room temperature (27 °C) for 30 min in the dark, and the absorbance was measured at 517 nm using a multimode microplate reader (Synergy biotek HTX) equipped with Gen 5.3 software. The antioxidant activity of each sample was measured using the DPPH assay by comparing its ability to scavenge free radicals against a blank. The DPPH inhibition rate was calculated using the equation below.

$$DPPH\;inhibition\;rate=\left[\frac{\left(A_{0}-A_{1}\right)}{A_{0}}\right]*100$$

where A_0_ is the absorbance of the control without a sample (Blank), and A_1_ is the absorbance of the sample.

The IC50 value, which represents the concentration of the antioxidant required to inhibit 50% of the DPPH radicals, was determined by plotting the percentage of inhibition against the antioxidant concentration and performing a linear regression analysis. This value was then compared to the IC50 value of ascorbic acid, a standard antioxidant, to evaluate the sample’s antioxidant potency. Inhibitory concentration of the sample was required to reduce 50% of DPPH.

#### 2.7.3. Total Monomeric Anthocyanin Content

To determine the total anthocyanin content (*C*), the pH differentiation method was used as per the method stated by the AOAC Official Method 2005.02. Extracted samples were diluted with 0.025 M (pH 1) potassium chloride buffer and 0.4 M sodium acetate buffer (pH 4.5). The absorbance measurements were taken using a UV-Vis Romulus spectrophotometer at two wavelengths, 520 nm and 700 nm. The total anthocyanin content was calculated as cyanidin-3-glucoside equivalents as per the equation below.

$$C=\frac{A\times Mw\times Df\times 10^{3}}{\in \;x\;L}$$

where A is the absorbance,

$$(\mathrm{Absorbance})=[\left(A520-A700\right)\;pH\;1.0\;-\;(A520-A700)\;pH\;4.5$$

Mw = molecular weight (448.8 g/mol for cyanidin-3-glucoside), Df = dilution factor, 10^3^ = conversion from gram to milligram, ε = molar extinction coefficient, L × mol^−1^ × cm^−1^ (26,900 L/mol/cm for cyanidin-3-glucoside), and l = pathlength (1 cm). The results were presented as the dry weight of the sample.

#### 2.7.4. Characterization of Anthocyanin by LC-MS Analysis

For the phenolic compounds investigation, the method explained by Bashmil et al. [34] was used with some modifications. In brief, LC-UV-MS was used for the characterization of anthocyanins in banana inflorescence. The highly sensitive and high-resolution Shimadzu LCMS-8050 Triple Quadrupole LC-UV-MS/MS instrument in MRM mode with a PDA detector was used for the phenolic characterization using a reverse phase column (Kinetex EVO C18, 100 mm × 2.1 mm × 2.6 um). The PDA detector conditions were 500–600 nm wavelength at the spectrum resolution of 256. The mobile phase A was 10% (*v*/*v*) formic acid in water and the mobile phase B was 10% formic acid in a 60:40 (*v*/*v*) mixture of methanol and acetonitrile. Blank (solvent) samples and standards were prepared using LCMS grade water and filtered through a 0.22 µM filter before being loaded into inserts and injected into the instrument following the same procedure as the filtered banana inflorescence extracts. Cyanidin-3-O-Glucoside at different concentrations (50, 25, 12.5, 6.25, 3.125, 1.56 µg/mL) was used for the calibration curve for absolute quantification. Five microlitres of banana extract was injected at an average flow rate of 0.4 mL/minute for a duration of 10 min at 40 °C and 4800 psi. MS investigation was performed in automated mode. The machine control, data acquisition, and analysis were conducted using skyline 23.1.1.335 software. The raw data were initially recorded with a precision of four decimal places and rounded to one decimal place in the results.

### 2.8. Statistical Analysis

The statistical differences were determined using Minitab 21.1 software (Minitab, LLC, USA). Analysis of Variance (ANOVA) test and Turkey’s test were used to analyze the statistical difference. If *p* < 0.05, the results were considered as significantly different. Data were presented as the average mean ± standard deviation.

## 3. Results and Discussions

### 3.1. Morphology

The results show that banana inflorescence used in this study had an average weight of 1.2 kg with a length of 32 cm and a width of 12 cm. The colour properties are listed in Table 1. For this study, uniform banana inflorescences were chosen. According to the results, the cultivar (*Musa cavendish*) chosen for this study is generally larger than the other banana inflorescence cultivars reported in the literature. Musa cavendish belongs to the triploid AAA group, which gives it distinct characteristics, such as larger fruits and inflorescences. In contrast, other cultivars, including *Musa paradisiaca* [35] and *Musa sapientum* [35], belong to the ABB group, which generally produces smaller fruits compared to the AAA type. This size variation can also be attributed to different geographical and agronomic conditions.

The literature reports other banana inflorescence cultivars that have been grown in specific regions, such as *Musa paradisiaca* in India, *Musa* AAB cv Prata Ana in Brazil, and *Musa* ABB cv Namwa in Thailand. Since this study was conducted in Australia—an area that has not been extensively studied—morphological characterization may provide valuable insights for future research.

The moisture content and ash content are in line with the proximate composition analysis reported for fresh *Musa sapientum* L. light yellow banana bracts, which is 93.92% ± 0.03 moisture and 0.58% ± 0.08 ash.

The moisture content reported in the present study is greater (Table 1) compared to the moisture level reported for the Cavendish cultivar which is 90.73% [23], the Baxijiao at 90.58% [35], and the Paradisiaca at 89.42% [35]. The moisture content also can vary due to many conditions such as geological conditions, season of harvesting, irrigation method, and genetic diversity. The colourimetric analysis was also carried out for Cavendish banana inflorescence as there is a lack of literature on the morphological characteristics of this inflorescence. The colour analysis will be a significant finding for future food product development with banana inflorescence as the colour is a dominant sensory property which evaluates consumer preference.

### 3.2. Microstructure

**Figure 5:**
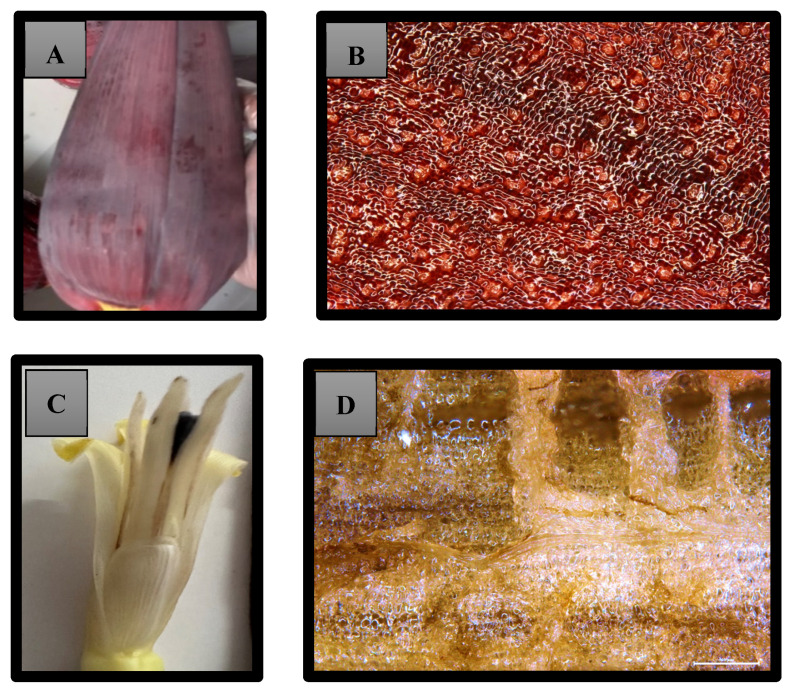
(**A**) Banana inflorescence bract, (**B**) light microscopy view of bracts, (**C**) banana male flowers—magnification 5000×, (**D**) light microscopy view of male flowers— magnification 5000×.

The microscopic analysis demonstrates the morphological characteristics of fresh samples used for the extraction. The microstructure of banana inflorescence bracts exhibits a dense arrangement of cells and very soft swollen cells. This shows the high moisture content of cells. No ruptured cells were observed before the extraction process. As the dead exposed outer bracts were removed at the pre-cleaning stage, the cells in the inner bracts used for the experiments demonstrated their precise cell structure and cell composition without any decomposition and degradation. The cell walls are also hard, rigid, well shaped, and consistent in the microscopic images, as shown in Figure 5. This may be due to the presence of higher amounts of fibres which gives a shape to the cells. The colour of the cells is red to purple which exhibited the availability of pigments such as anthocyanins in banana inflorescence cells. The male flowers exhibited non-colourant properties in light microscopic images. However, this shows the presence of higher moisture content and softer cells, which aids in efficient bioactive compounds extraction.

### 3.3. Total Phenolic Content (TPC)

The extracted banana inflorescence exhibited a substantial amount of phenolic content. Table 2 compares the Folin–Ciocalteau experimental data on the total phenolic content of banana inflorescence at different temperatures and solvent mixtures. As per Table 2, the results show the availability of phenolic compounds in fresh banana inflorescence. All the solvent mixtures (water, methanol, ethanol) at all temperature levels (60 °C, 80 °C, 100 °C) showed their potential in extracting phenolic compounds from the banana inflorescence. This research shows for the first time the extraction of polyphenols from banana inflorescence. Furthermore, the results show the compatibility of banana inflorescence with the ASE method.

There was a significant difference (*p* < 0.05) in the total phenolic compounds resulting between treatments. Another important finding is that the highest concentration of phenolic compounds in banana inflorescence resulted in the highest temperature. Banana inflorescence extracted at 100 °C with accelerated solvent extraction was greater than the extracts at 80 °C and 60 °C. According to the findings exhibited in Table 2, the lowest phenolic content resulted in the extraction at 60 °C, and the highest phenolic content resulted at 100 °C. Previous studies on banana inflorescence have reported the total phenolic content only at low-temperature ranges between 50 °C and 80 °C [23,36]. The current findings showed that the total phenolic content at 100 °C is between 725.7 ± 2.0 and 1239.6 ± 20.8 mg/100 g. However, this finding is in agreement with the previous results on ultrasound-assisted banana inflorescence phenolic compound extracts explained by Schmidt et al. [37] which shows the positive relationship between the temperature of extraction and the total phenolic content of the extract.

Extractions at elevated temperatures resulted in a higher yield of antioxidants and total phenolic compounds. This can be explained by the elevated temperatures which were higher than the boiling points of the solvents, increasing the diffusion rates, disrupting the solvent and matrix interactions, reducing solvent viscosity, decreasing the surface tension of the solvents, and improving the penetration of the solvents into the cells, and this resulted in the rapid extraction of the bioactive compounds by consuming fewer solvents. This finding is in agreement with the ASE of black sorghum [38] which resulted in a higher yield of phenolic compounds and antioxidants at 120 °C and 150 °C compared to 60 °C. Another recent study conducted on Tahiti lime pomace is also in agreement with the conclusion that higher temperatures improved the antioxidant activity as the total phenolic content examined at 110 °C was greater than the yield at 60 °C and 85 °C [29].

**Figure 6:**
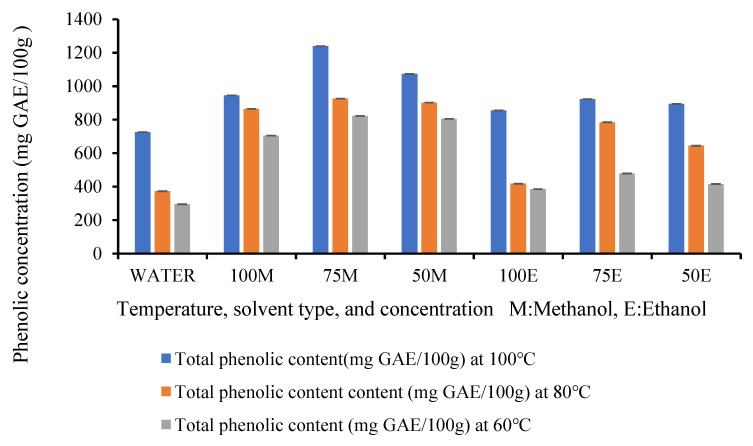
Effect of extraction parameters on the total phenolic compounds analysis.

**Figure 7:**
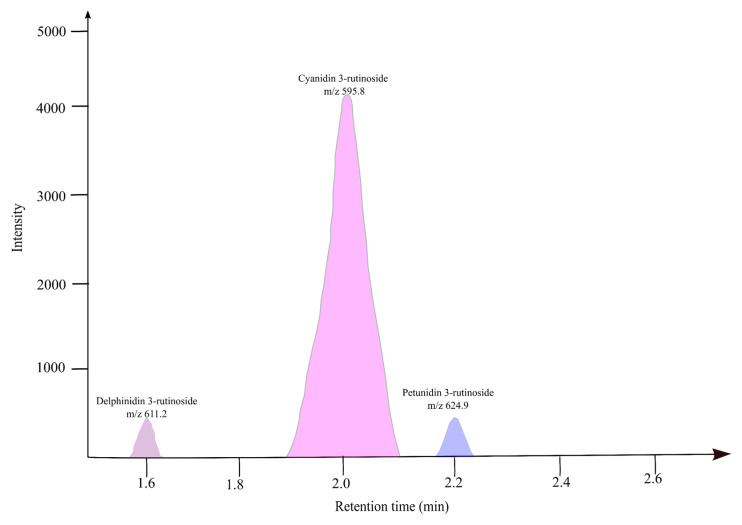
Anthocyanin profile of banana inflorescence-LC-MS/MS chromatogram.

As shown in Figure 6, the results further demonstrate that there is a significant effect of solvent on the total phenolic content. All the solvents including water, methanol, and ethanol were capable of extracting phenolic compounds from banana inflorescence; however, these results were significantly different, demonstrating that the solvent type has a significant impact on the efficiency of the extraction of phenolic compounds. According to the results shown in Figure 7, methanol was superior to water and ethanol at all three temperature levels of extraction examined in this study. Water was the least compatible with phenolic extraction from banana inflorescence. Most of the literature on investigating phenolic compounds concluded that methanol is the best solvent for phenolic extractions [39]. This study is also in agreement with the previous findings. The highest efficiency of extraction by methanol could be attributed to the properties of methanol. During the dissolution process, the OH bonds of phenol compounds effectively form H bonds with methanol rather than ethanol and water. Further, methanol is considered a solvent that can extract a wide range of compounds with different polarities [40].

The impact on solvent concentration was also investigated in this experiment. The findings showed that 75% methanol in an aqueous solution was the best solvent concentration for phenolic compound extraction from banana inflorescence. This could be due to the increasing polarity of the aqueous methanolic mixture; 75% methanol is the highest because the mixture of methanol and water enhances the extraction efficiency. Water in the 75% methanol solution helps in breaking the plant cell walls, releasing more phenolic compounds. Additionally, many phenolic compounds are polar in nature, and a mixture of methanol and water creates an optimal polarity that improves their solubility. In contrast, 100% methanol lacks the polarity balance provided by water, which can limit the extraction of certain phenolic compounds, resulting in a lower concentration. Optimizing the extraction processes is critical for advancing future research, particularly in enhancing energy efficiency. The identification of optimal conditions allows for the preservation of phenolic content while minimizing both solvent concentration and extraction time. Since both time and solvent usage significantly influence the energy costs associated with extraction, refining these parameters is essential. The development of optimized extraction methods offers the potential to substantially reduce these costs, contributing to more sustainable and energy-efficient practices in future studies.

The total phenolic content of fresh Cavendish banana inflorescence ranges between 295.14–1239 mg GAE/100 g for dry basis in this present experiment. This finding is greater than the reported phenolic concentration (201 mg/100 g) for *Musa* sp. cv. Nanjangud rasa bale was processed via oven drying at 40 °C followed by Soxhlet extraction [41]. These variations in total phenolic content could be mainly due to the use of different varieties, cultivars, different solvents for extraction, and the choice of processing method. The current result is slightly below the range of phenolic content reported by Schmidt et al. [37] using the same Folin–Ciocalteau test for oven dried (55 °C) Cavendish banana inflorescence extracted with 50% ethanol at 60 °C. This study reported a total phenolic concentration of 1690 mg GAE/100 g. Another study reported a higher phenolic concentration of 22.74 mg GAE/g for shade dried whole *Musa paradisiaca* extracted via maceration using ethanol as a solvent [42]. The current experiment was carried out on fresh samples and the previous literature reported phenolic concentration for dried samples. During the drying steps and extraction procedures, the total phenolic composition may have increased compared to the fresh samples by enhancing cell breakage and inducing the extraction of polyphenols.

However, the use of fresh plant samples in food production offers significant advantages in terms of energy savings compared to dried samples. Drying processes, which are commonly employed to extend shelf life and preserve bioactive compounds, are energy-intensive, often requiring substantial heat and extended processing times. This not only increases the overall energy consumption but also adds to the environmental impact and operational costs of production.

In contrast, fresh plant samples eliminate the need for drying, thereby reducing the energy input associated with the production process. Utilizing fresh materials also ensures the retention of moisture content, which can be beneficial for maintaining the quality and nutritional properties of the final product. Thus, adopting fresh plant samples in food production can contribute to both economic efficiency and environmental sustainability by minimizing energy usage.

### 3.4. Antioxidant Activity

Antioxidant activity or antioxidant potential is determined by IC50 values. The IC50 value indicates the concentration of antioxidant ions required to reduce the free radical DPPH by half of the initial concentration. Briefly, aliquots of 100 µL of the extracts were mixed with 100 µL of DPPH 0.01 M solution. The mixtures were vortexed and incubated at room temperature in the dark for 30 min. Subsequently, the absorbance was measured via a spectrophotometer at 517 nm. The lower the IC50, the greater the antioxidant activity. According to the findings given in Table 2, there was a significant difference between treatments for the IC50 values, indicating significant differences in the antioxidant activities of the extracts. There was a positive correlation between the total phenolic content and IC50 values. The highest antioxidant potentials were recorded for the extracts with the highest phenolic concentrations. This proves that phenolic compounds are responsible for the antioxidant activity of banana inflorescence. This finding is in accordance with previous results from Schmidt et al. [23] reported for the dried *Musa cavendishii* banana inflorescence, and also the study on a Thai food made from banana inflorescence [11].

The present results conclude that 75% methanol extracted at 100 °C is superior in terms of antioxidant activity to other extracts followed by 50% methanol and 100% methanol extracted at the same temperature. The extraction temperature showed a significant correlation with antioxidant activity. The antioxidant activity of the extract obtained at 100 °C was superior to that obtained at 80 °C and 60 °C. As per the results, it can be concluded that the increased temperature of ASE resulted in increased antioxidant activity of the extract. This can be further explained as the rise in temperature accelerates the mass transfer, enhancing the efficiency of cell wall breakages, and increasing the solubility and diffusion of chemicals [23]. The antioxidant results further confirmed that the extraction solvent composition highly influences the antioxidant activity. According to the findings, methanol was superior to the other solvent mixtures. ASE allows the solvents to remain liquid at elevated temperatures which increases the mass transfer and solubility. ASE improves the preservation of heat-sensitive compounds and reduces their degradation rate during the extraction process.

The results have demonstrated that the elevated temperatures resulted in higher quantities of bioactive compounds for all the solvent mixtures. Similar findings were reported from recent research on Tahiti lime utilizing accelerated solvent extraction. The elevated temperature resulted in significantly higher concentrations of antioxidant and phenolic compounds. The results suggest a strong correlation between temperature and the extraction of bioactive compounds. Further studies are recommended to optimize extraction conditions [29]. Another study on grape pomace also concluded that ASE enhances phenolic extraction at temperatures above 100 °C [31] without degradation of anthocyanins.

### 3.5. Total Monomeric Anthocyanin Content

The total anthocyanin content was recorded as 22.82 ± 1.91 mg/100 g (DW) via the pH different method for fresh Cavendish banana inflorescence The anthocyanin content of dried *Musa ABB* culinary banana inflorescence was reported as 58 mg/100 g [8,15,43], which is greater than our results. This could be due to the cell wall disruption during the drying procedure. However, the anthocyanin content for Cavendish banana inflorescence for the fresh or dried samples has not previously been compared. Therefore, this study contributed to filling the knowledge gap in this research area.

Investigating the anthocyanin content of fresh banana inflorescence using accelerated solvent extraction (ASE) is highly significant for both energy savings and sustainability [31]. In addition to energy efficiency, ASE optimizes solvent use by requiring smaller quantities, further minimizing environmental impacts. The ability to extract anthocyanins in a more sustainable manner enhances the potential for creating high-value products from banana by-products, which aligns with circular economy principles. Overall, this method promotes both sustainable resource use and energy conservation in food and nutraceutical production, supporting broader sustainability goals.

### 3.6. Anthocyanin Profile of Fresh Banana Inflorescence Extract

Anthocyanins are important bioactive compounds present in many fruits and vegetables including blueberry, red cabbage, chokeberry, strawberry, raspberry, blackcurrant, cherry, and brinjal, with superior antioxidant potential [44]. The anthocyanin profile of dried Musa ABB culinary banana inflorescence was determined by HPLC -MS/MS. Banana bracts are rich in three different anthocyanidins, cyanidin-3-o-glucoside (0.88 mg/100 g), yanidin-3-o-rutinoside (4.06 mg/100 g) and Peonidin-3-o-glucoside (3.69 mg/100 g). 

An LC-MS chromatogram of banana inflorescence extracted at 75% methanol at 100 °C revealed three major peaks, shown in Figure 7. The retention time, molecular *m*/*z* fragment *m*/*z*, and the name of the compounds are given in Table 3. The results confirmed that the anthocyanin core is attached to the rutinosyl group.

The first prominent peak was recorded at the retention time (RT) of 1.6 min, the molecular *m*/*z* was 611.2, and it resulted in the fragment ion at 302.7 which is confirmed as delphinidin. The second peak resulted at the 2.0 min RT, 595.8 molecular *m*/*z*, and the fragments *m*/*z* at 287.7 were identified as cyanidin. This cyanidin peak was more prominent in the chromatogram. The third peak resulted at the 2.2 min RT, 624.9 molecular *m*/*z*, and the fragments *m*/*z* at 317.7 were identified as petunidin.

Table 3 shows the concentration of the anthocyanidin compounds identified as % of total anthocyanin and the concentration as a cyanidin equivalent. The MS results exhibited higher concentrations of Dp, Cy, and Pt anthocyanidins. The concentrations of these anthocyanins are 0.082 µg/mL, 1.546 µg/mL, and 0.082 µg/mL for Dp, Cy, and Pt, respectively. This is equivalent to 90% Cy and 5% Dp and Pt equally.

Delphinidin is detected with a rutinoside group as per the chromatogram of anthocyanidins found in fresh banana inflorescences. DP is responsible for different coloured plant pigments as well as medicinal functions in human, plant, and animal cells [45]. These compounds possess antioxidant, anticancer, and antidiabetic properties, as per previously published studies [46].

**Figure 8:**
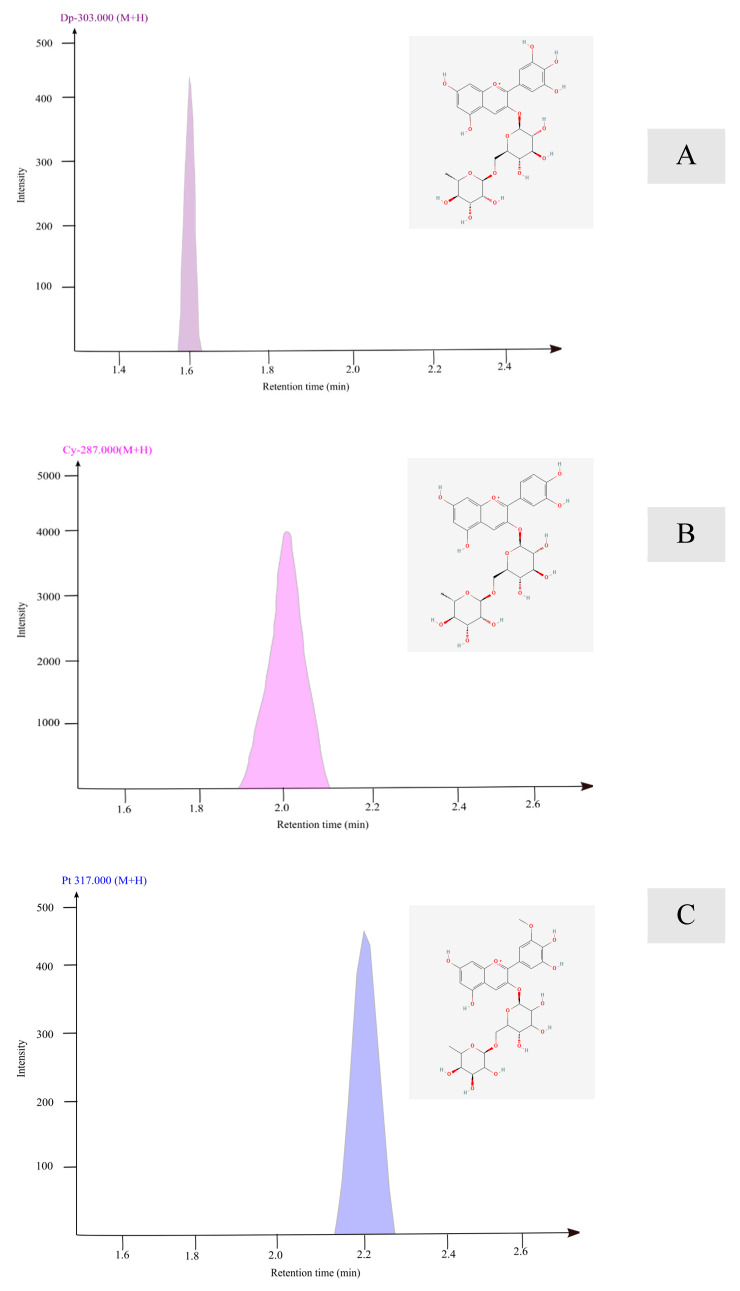
LC-MS/MS chromatogram and the chemical structure of (**A**) Dp-3-rutinoside; (**B**) Cy- -3-rutinoside; and (**C**) Pt-3-rutinoside.

According to the chromatogram, the most prominent peak is identified as Cyanidin-3-rutinoside, which is responsible for the orange-red colour pigment as shown in Figure 8B. The other identified compounds were Dp and Pt. DP and Pt are accountable for the blue-red colour pigments with slight colour variations. The LC-MS/MS results showed that more than 90% of anthocyanin available in fresh banana inflorescence is Cy. This finding is in agreement with most anthocyanin-rich fruits and vegetables including red-purple colour berries [47]. According to the findings, banana inflorescence was found to be a great source of Cy-3-rutinoside. Cy-3-rutinoside is an anthocyanin, a glycoside, and a rutinoside derivative. Cyanidin-3-rutinoside is a natural polyphenolic extract, one of the most widespread anthocyanidins with a positive health impact on humans and animals. The therapeutic potential of Cy includes reducing antioxidative stress, anti-inflammatory, chemoprotective, anti-aging, cardiovascular-protective, antidiabetic, nervous-protective, and antitoxic activity [48].

Petunidin is the third anthocyanin type discovered from fresh banana inflorescence at a retention time of 2.2 as illustrated in Figure 8C. Petunidin is also connected with the rutonosyl group. Pt is identified as a strong antioxidant under the anthocyanin group. It is widely known for its contrasting colour. The therapeutic benefits of Pt are significantly important for the prevailing non-communicable diseases around the world. Pt has shown chemoprotective capacity, reduces the risk of heart problems, and is an excellent candidate for pharmaceutical development [49]. Petunidin is detected in fruits and vegetables such as blackcurrants, black grapes, blueberries, bilberries, and black beans [49,50]. However, the availability of petunidin in waste Cavendish banana inflorescence has not been discovered in the literature. Therefore, this finding is an innovative finding that supports waste valorization as well as future therapeutic product development.

Moreover, the anthocyanidins profile in the fresh banana inflorescence of the Cavendish cultivar has not been studied before. Therefore, this study provides the first analysis of the anthocyanidins present in the fresh Cavendish banana inflorescence. The findings of this study can be further extended to the isolation and purification of anthocyanidins for commercial purposes, such as the production of food colourants rich in antioxidants. These results have significant implications for the food and pharmaceutical industries, and further research in this area is warranted in agreement with previous findings reported in the literature by Yücetepe et al. [51].

## 4. Conclusions

This study has conducted an experimental procedure for extracting health-promoting bioactive compounds from fresh banana inflorescence using a sustainable extraction method. Accelerated solvent extraction (ASE), using optimized solvent composition and conditions, enabled higher yields of anthocyanins with minimal degradation, preserving their bioactivity. This finding can contribute significantly to future research studies on green extraction techniques, as the previous literature only examined conventional extraction methods. This study has validated the sustainable approach to valorizing banana inflorescence and its potential in bioactive compound extraction.

Furthermore, this study has revealed that fresh banana inflorescence contains an abundance of bioactive compounds that can be extracted for value-added product development in the pharmaceutical and nutraceutical industries. The availability of these bioactive compounds in fresh banana inflorescence presents a great opportunity for enhancing new income opportunities in banana farms with minimal processing requirements, low technical skills, and low production costs. Additionally, fresh banana inflorescences can also be utilized directly in the extraction process, thus reducing energy demand and time consumption.

The biorefinery process of banana inflorescence contributes to all three pillars of sustainability: social, environmental, and economic. Therefore, this study directly contributed to the United Nations’ sustainable development goals (SDGs). The extraction, isolation, and identification of pharmacologically important compounds from waste by-products for value-addition purposes is contributing to SDG 3, “Good health and well-being”; SDG 12, “Responsible consumption and production”; and SDG 9, “Promotion of industrialisation, innovation, and infrastructure”.

## Figures and Tables

**Table 1:** Morphological characteristics of fresh banana inflorescence.

Morphology	Fresh
Length (cm)	32 ± 4.00
Width (cm)	12 ± 3.00
Weight (Kg)	1.23 ± 0.97
Moisture %	92.96 ± 0.15
Ash content %	0.59 ± 0.01
Colour—HEX (#)	533B3C
Colour (L*)	27.46 ± 0.05
a*	11.13 ± 0.11
b*	3.53 ± 0.11
c*	11.8 ± 0.24
h*	17.6 ± 0.21

**Table 2:** Total phenolic content and scavenging activity of the extracts collected at different temperatures using different solvent mixtures.

Temperature	Solvent Type	Total Phenolic Content (mg GAE/100 g DW)	DPPH Scavenging Activity (IC 50) mg/mL
100 °C	Water	725.7 ± 12.0 ^h i^	3.70 ± 0.02 ^a b c d e^
	Methanol	945.6 ± 21.2 ^c^	2.58 ± 0.03 ^i^
	75% Methanol	1239.6 ± 20.8 ^a^	2.21 ± 0.03 ^j^
	50% Methanol	1073.1 ± 18.3 ^b^	2.45 ± 0.05 ^i j^
	Ethanol	855.3 ± 39.4 ^o f^	3.47 ± 0.11 ^d e f g^
	75% Ethanol	923.2 ± 6.8 ^c d e^	3.27 ± 0.05 ^a b c d e^
	50% Ethanol	894.1 ± 10.9 ^c d e^	3.37 ± 0.03 ^f g^
80 °C	Water	372.8 ± 14.3 ^l^	3.91 ± 0.01^a b^
	Methanol	864.6 ± 20.8 ^d e f^	3.44 ± 0.03 ^e f g^
	75% Methanol	926.3 ± 19.7 ^c d^	2.93± 0.04 ^h^
	50% Methanol	902.4 ± 29.4 ^c d e^	3.37 ± 0.04 ^f g^
	Ethanol	417.5 ± 19.4 ^k l^	3.82 ± 0.05 ^a b c^
	75% Ethanol	784.7 ± 26.2 ^g h^	3.60 ± 0.03 ^b c d e f^
	50% Ethanol	644.6 ± 35.5 ^j^	3.74 ± 0.02 ^a b c d e^
60 °C	Water	295.1 ± 12.0 ^m^	3.95 ± 0.02 ^a^
	Methanol	704.9 ± 31.8 ^i j^	3.72 ± 0.02 ^a b c d e^
	75% Methanol	822.9 ± 20.8 ^f g^	3.51 ± 0.05 ^c d e f g^
	50% Methanol	804.86 ± 25.12 ^f g^	3.53 ± 0.01 ^c d e f g^
	Ethanol	385.4 ± 20.8 ^l^	3.90 ± 0.05 ^a b^
	75% Ethanol	478.8 ± 10.4 ^k^	3.76 ± 0.04 ^a b c d^
	50% Ethanol	415.9 ± 10.5 ^k l^	3.47 ± 0.14 ^c d e f g^

Values are the mean ± standard deviation, and those with different letters are significantly different (*p* < 0.05).

**Table 3:** Identification of anthocyanin profile of fresh banana inflorescence via LC-MS.

Peak	Retention Time (min)	Molecular (*m*/*z*)	Fragments (*m*/*z*)	Compound Name	Anthocyanin%	Concentration as Cyanidin Equivalent µg/mL
1	1.6	611.2	302.7	Dp-3-rutinoside	4.90 ± 0.01	0.082
2	2.0	595.8	287.7	Cy-3-rutinoside	90.03 ± 0.07	1.546
3	2.2	624.9	317.7	Pt-3-rutinoside	4.90 ± 0.05	0.082

## Data Availability

The original contributions presented in the study are included in the article, further inquiries can be directed to the corresponding author.
